# Effects of street greenery and seasonality on health and wellbeing of cyclists: a Virtual Reality experiment

**DOI:** 10.1038/s41598-026-64499-4

**Published:** 2026-07-29

**Authors:** Silviya Korpilo, Omkaranathan Ravindran, Jussi Torkko, Elias Willberg, Sini Ruohomäki, Elina Numminen, Ashish Shete, Ann Ojala, Tuuli Toivonen

**Affiliations:** 1 Digital Geography Lab, Department of Geosciences and Geography, University of Helsinki, Helsinki, Finland; 2 Helsinki Institute of Sustainability Science, University of Helsinki, Helsinki, Finland; 3 Helsinki Institute of Urban and Regional Studies, University of Helsinki, Helsinki, Finland; 4 Department of Mathematics and Statistics, Faculty of Science, University of Helsinki, Helsinki, Finland; 5 Natural Resources Institute Finland, Helsinki, Finland

**Keywords:** Virtual reality, Green infrastructure, Seasonality, Health and wellbeing, Active mobility, Urban planning, Environmental sciences, Psychology, Psychology

## Abstract

Street vegetation can play an important multifunctional role in increasing green spaces for more just, healthy and sustainable cities. However, there is still limited knowledge on how greenery exposure and seasonal conditions affect health and wellbeing during everyday active travel. This study integrates Virtual Reality, biosensors and visual semantic segmentation to test and compare different green infrastructure scenarios: only built, low greenery, high greenery and a winter condition. Using a between-subjects design, 138 participants were randomised to cycle in one of the four conditions, while gathering data on various physiological (skin conductance, blink rate, pupil diameter) and psychological outcomes (self-reported pre and post measures of restoration, affective states, subjective vitality). The results provide important empirical evidence that street greenery can decrease negative emotions and provide momentary stress reduction even under rapid exposure during a cycling commute. The findings also demonstrate high reactivity to natural elements in virtual environments.

**Supplementary Information:**

The online version contains supplementary material available at https://doi.org/10.1038/s41598-026-64499-4.

## Introduction

Current policies such as the UN’s Biodiversity Plan for Life on Earth and the EU 2024 Nature Restoration Law set ambitious goals for preserving, restoring and increasing green spaces for more just, healthy and sustainable cities^1,2^. As urban areas continue to densify, it is crucial to understand how small-scale green infrastructure such as street vegetation can provide a feasible solution for achieving these aims.

Street greenery interventions provide untapped opportunities to balance socio-ecological needs in a limited space. In addition to increasing overall green cover in cities, street greenery has been found to contribute to active mobility in several ways: by increasing cycling uptake, frequency and volume^3–5^, by promoting pleasantness and satisfaction^6–9^, by providing restoration and mental health benefits^10,11^, and by reducing adverse environmental exposures such as heat and noise, while increasing exposure to natural sounds and higher soundscape quality^12,13^. However, there is still limited knowledge on the causal mechanisms of health and wellbeing effects during travel, and how they are affected by different street greening designs^14,15^. This is caused primarily by the dominant residential-based and recreational perspectives in nature-health research^16^, the lack of consensus of what is considered street greenery, and the diverse methods used to study greenery exposure effects^15,17,18^. There is also limited knowledge of how seasonal variations in greenery exposure affect health and wellbeing during active mobility^11,19^.

To fill these gaps, this experimental study examines the immediate effects of different greenery and seasonal conditions on health and wellbeing of cyclists during everyday travel. We employed Virtual Reality (VR) as a novel and immersive method to study causal effects of environmental stimuli in laboratory settings. Increasingly used in the nature exposure and health domain, VR builds on a long tradition of nature simulation studies that originated in the 1970s to examine planning and management of forests, rural and recreational areas^20,21^. VR technologies facilitate control and customisation over designs and environmental conditions, reduce confounding effects, and save time and resources needed in real-world interventions such as allowing for vegetation to grow naturally or replicating assessments in multiple seasons. Virtual environments also provide greater immersion and sense of presence compared to traditional methods such as 2D photos or videos^22^.

The use of VR in various applications has been growing rapidly including nature exposure therapy and pain management^23–25^, psychological restoration^26–30^, green exercise^31^, nature connectedness^32^, environmental preferences^33,34^, and walking and cycling behaviour^35–38^. Recent literature reviews show that findings vary across VR designs and methodological approaches, but generally indicate psycho-physiological benefits of virtual nature, especially related to improved mood, attention and cognition, and perceived restorativeness and stress reduction^35,39–41^. However, mobility approaches in VR have remained limited, especially in relation to cycling^36,39,41^. In terms of environmental elements, there is significant evidence on the positive effects of greenery and vegetation, but previously less attention has been given to different environmental conditions or diversity of vegetation^27,30,35^.

Therefore, to contribute to the scarce literature on the effects of green infrastructure on active mobility, we developed and tested four future VR scenarios with different computer-generated street environments: (1) built (control, almost no vegetation, ambient traffic noise); (2) low level of greenery (low amount and diversity of vegetation, ambient traffic noise, few birdsongs); (3) high level of greenery (higher amount and diversity of vegetation, ambient traffic noise and more birdsongs); and (4) winter condition of environment (3). We compared the effects of these four exposure conditions and tested three main hypotheses that are based on previous evolutionary and theoretical perspectives of the biophilia hypothesis^42^, Stress Reduction Theory^43^, Attention Restoration Theory^44^, as well as the more recent greenery hypothesis^45^. These theories propose that humans have an inherent affinity for nature and that even brief or indirect exposure to natural elements can reduce stress and restore depleted cognitive resources. Therefore, we first hypothesise that street greenery promotes psycho-physiological restoration (i.e. reduces physiological stress and increases self-reported restoration) and improves mood (i.e. increases positive affect and reduces negative effect) and subjective vitality during cycling. Our second hypothesis is that these effects will be strongest for the VR environment with high level of greenery, which has higher auditory-visual diversity, compared to the low greenery and only built infrastructure environments. Since VR and active mobility studies focusing on seasonality are still scarce and findings have been mixed^35,46^, our final hypothesis is exploratory, postulating that there is a difference in the restoration and wellbeing effects between the summer and winter environments.

Since most previous empirical studies still lack comprehensive psycho-physiological integration^47^, here we test the theoretical rationale by combining three psychological (perceived restoration, affective states, and subjective vitality) and three physiological measures (skin conductance, blinking rate, and pupil diameter). While the psychological measures and skin conductance are well-established in the field^47^, the latter two are less studied eye-tracking biomarkers that can be used as indicators of emotion and attention restoration. Typically, eye blinking increases during moments when the probability of missing important visual information is low, indicating less directed attention and engagement^48,49^. Pupil diameter is used to measure pupil size which naturally adjusts to light conditions but can change independently of changes in luminance reflecting internal cognitive processes. Pupil dilation is also a marker of emotional stimuli, acute attention and cognitive load^48,50^. Overall, our aim is to advance psycho-physiological studies on mobility exposure by using emerging technologies and biophysical indicators.

## Results

### Overall differences between the VR cycling environments

**Fig. 1:**
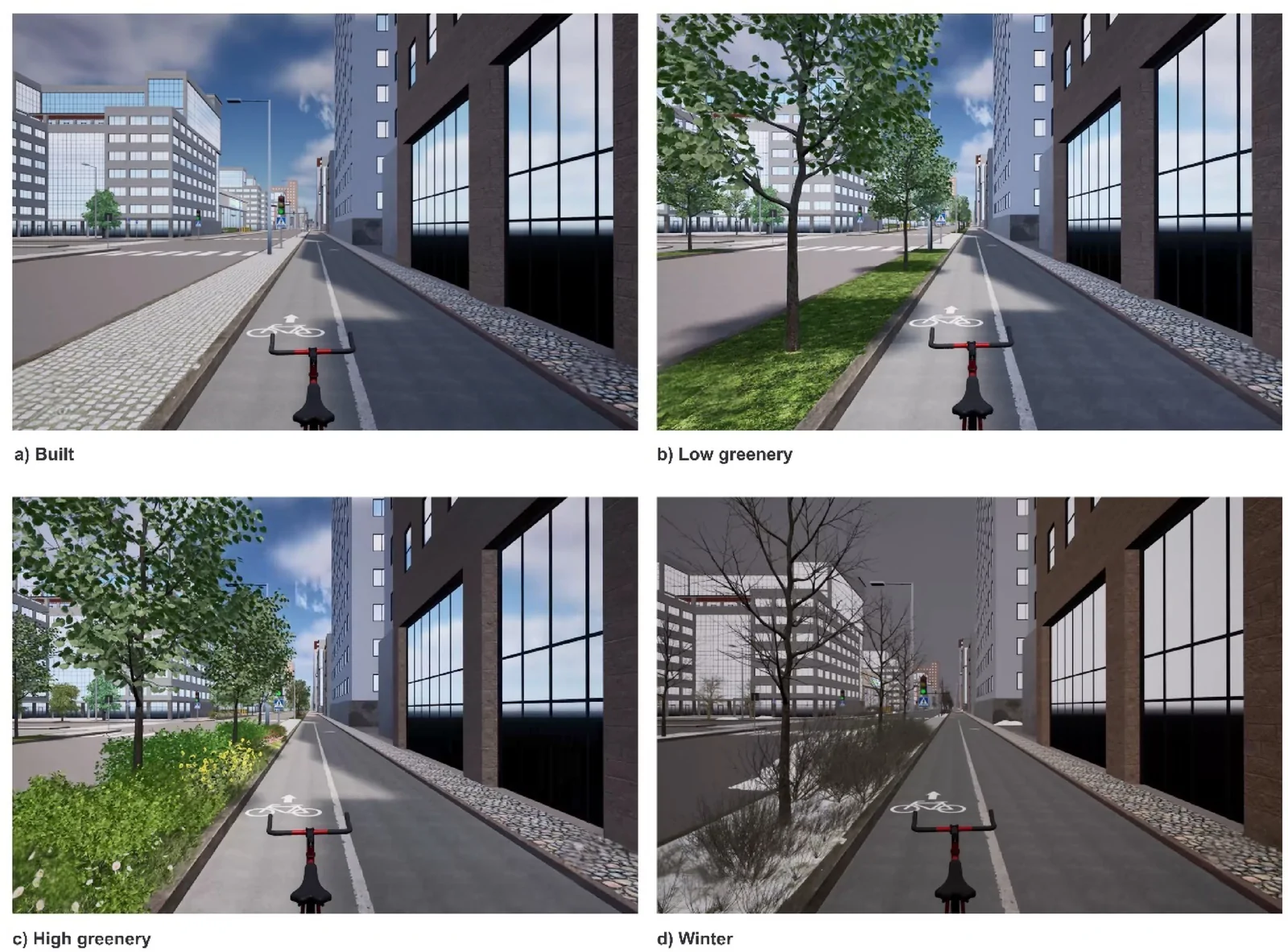
The four Virtual Reality cycling environments used in the study: built, low greenery, high greenery, and winter.

**Fig. 2:**
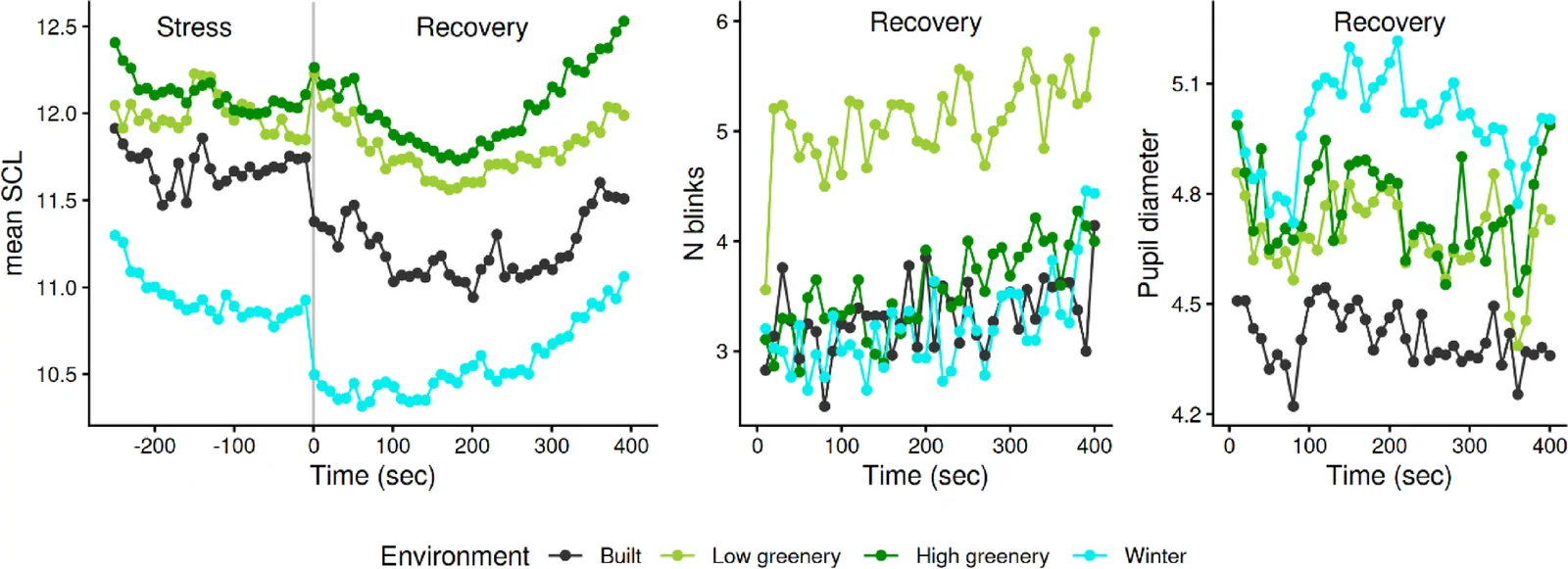
Mean Skin Conductance Levels (SCL) (µSiemens) in the Stress (sitting, no VR) and Recovery (cycling in VR) periods for participants in the built, low greenery, high greenery and winter environments. Number of blinks and pupil diameter (mm) were measured only in the Recovery period.

After a stressful mental arithmetic task, 138 participants were blindly randomised to one of the four recovery conditions: built (*N* = 30), low greenery (*N* = 34), high greenery (*N* = 39) or winter (*N* = 35) (Fig. 1). Individual greenery exposure values were calculated based on the Green View Index (GVI) of participants’ recorded VR headset videos, which changed according to how much people looked around while cycling. Mean GVI values of the environments were statistically different from each other: Built µGVI = 0.001; Low greenery µGVI = 0.221; High greenery µGVI = 0.282; Winter µGVI = 0.064 (ANOVA F = 1152.54, *p* < 0.001, Tukey’s post-hoc *p* < 0.001). In relation to the outcome measures, participants exhibited the highest average skin conductance level (SCL) in the high greenery group both during the stress and recovery periods, and participants in the winter group had the lowest average SCL values, but mean values between groups did not differ significantly (Table 1; Fig. 2).

**Table 1:** Mean and standard deviation (SD) of the physiological measures (mean/10 sec) during recovery period: Skin Conductance Level (µSiemens); Number of blinks; Pupil diameter (mm); and change in pre-post psychological measures: Restoration Outcome Scale (ROS), Positive and Negative Affect subscale (PANAS-POS and PANAS-NEG), Subjective Vitality Scale (SVS).

Environment	SCL (µS) Mean ± SD	N blinks Mean ± SD	Pupil dm (mm) Mean ± SD	ROS Mean ± SD	PANAS -POS Mean ± SD	PANAS-NEG Mean ± SD	SVS Mean ± SD
Built (N = 30)	11.4 (± 5.47)	3.16 (± 1.97)	4.37 (± 0.71)	1.53 (± 0.92)	−0.026 (± 0.57)	−0.086 (± 0.40)	−0.052 (± 0.88)
Low greenery (N = 34)	11.8 (± 4.55)	4.95 (± 2.93)	4.67 (± 0.77)	1.57 (± 0.83)	0.165 (± 0.53)	−0.188 (± 0.26)	0.228 (± 0.80)
High greenery (N = 39)	12.14 (± 4.15)	3.43 (± 1.83)	4.79 (± 1.11)	1.22 (± 0.78)	0.037 (± 0.58)	−0.119 (± 0.29)	0.211 (± 0.79)
Winter (N = 35)	10.5 (± 3.95)	3.11 (± 1.75)	4.99 (± 0.87)	1.22 (± 0.94)	−0.062 (± 0.52)	−0.053 (± 0.43)	0.154 (± 0.87)
ANOVA (p)	0.512	0.002*	0.054*	0.183	0.353	0.444	0.539

When averaging over the entire recovery period, there were significant differences in the mean blink rates of participants in the four conditions (*p* = 0.002). Participants blinked most frequently in the low greenery environment (µblink rate = 4.95) and least in the winter environment (µ = 3.11). In addition, the mean pupil diameter was significantly different between environments (*p* = 0.054). As expected, mean pupil diameter was largest in the winter environment (µ = 4.99 mm), which indicates pupil dilation to adjust to darker light conditions. Pupil diameter was smallest (µ = 4.37 mm) in the built environment, in which the lack of vegetation may have caused higher brightness conditions.

**Fig. 3:**
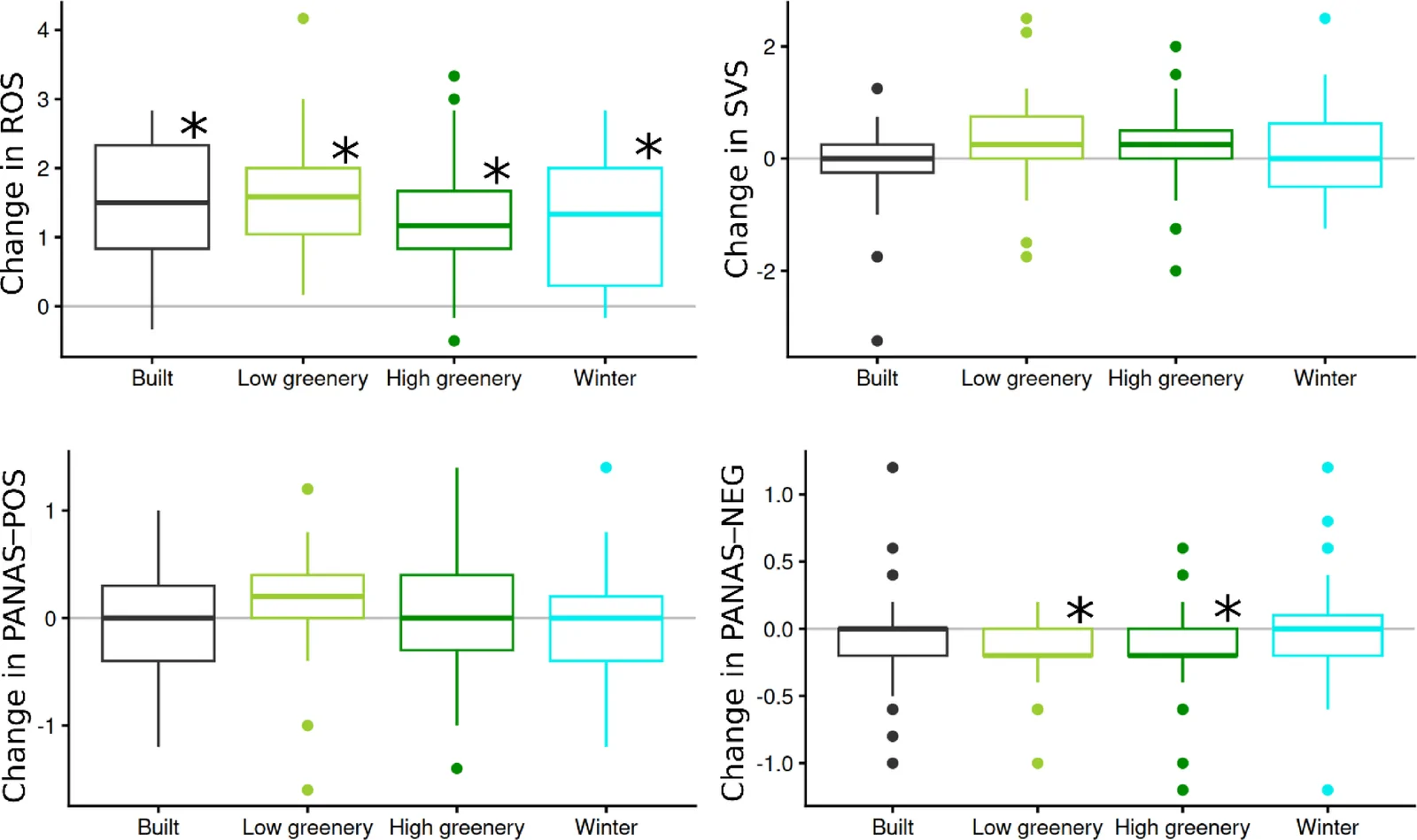
Change in self-reported psychological measures (pre-post questionnaire). ROS: Restoration Outcome Scale; SVS = Subjective Vitality Scale , PANAS-POS and PANAS-NEG = Positive and Negative Affect subscale. Bonferroni corrected significant paired t-test results highlighted with *.

In relation to changes in psychological measures, self-reported restoration (ROS values) significantly increased in all conditions in the post-questionnaire compared to pre-questionnaire (*p* < 0.001) (Fig. 3; Table 1 in Supplementary). Increase in positive affect (PANAS-POS) was reported for the low and slightly for the high greenery environments, although the changes were non-significant. However, there was a significant decrease in self-reported negative affect (PANAS-NEG) for participants in both greenery conditions: low (*p* = 0.003) and high (*p* = 0.04). There was also an increase in self-reported subjective vitality (SVS) for all conditions except the built, which showed slight decrease in mean scores, but the difference in pre-post measures was non-significant.

### Main effects on psycho-physiological outcomes

First, we tested for overall effects of the four environments on the mean psychological (ROS, PANAS-POS, PANAS-NEG, SVS) and physiological (adjusted to baseline SCL, blink rates, pupil dm) outcomes with a regression model. The model included individual background variables (gender, age, stressful day, self-reported physical and mental health, nature connectedness, cycling frequency) as covariates to control for the potential effects on the outcomes (Table 1; for full model results, see Supplementary 2). When controlling for individual background variables, results from the regression models indicated very few significant variables that would explain the psychological outcomes. In general, no significant effect of the environment type was found, but there was a significant relationship between better self-reported mental health and increase in perceived restoration (*p* = 0.005) (Table 2).

The findings for the physiological variables were more evident, especially for the eye-tracking data (Table 2). The environment type had no statistically significant overall effect on mean SCL levels, except for the high greenery condition, which had marginally significant positive effect (*p* = 0.059). Increasing age was associated with lower mean SCL, which reflects natural age-related decline in electrodermal activity^51^. However, contrary to expectations, higher nature connectedness was associated with higher SCL (*p* = 0.030). When compared to the built environment, there was a positive and significant association of both winter and high greenery environments with increase in pupil diameter, and a significant positive association between the low greenery condition and increased blink rate. Older age was associated with decreased pupil diameter and increase in number of blinks, while being a male was associated with reduction in blinking (Table 2).

**Table 2:** The computed statistical significance (p-value) and the corresponding estimated effects (in parentheses) of environment type and individual background covariates on mean physiological and psychological (pre-post change) variables. Baseline factor levels: built environment, female gender, and 18–24 age group. ** *p* < 0.01, * *p* < 0.05.

Predictor	SCL	Blink rate	Pupil diameter	ROS	PANAS-POS	PANAS − NEG	SVS
Intercept	0.810 (0.233)	0.013 (4.297)	< 0.001 (4.958)	0.119 (−0.954)	0.814 (−0.101)	0.079 (−0.455)	0.612 (0.306)
Low greenery	0.151 (0.484)	0.007 (1.578)	0.079 (0.400)	0.083 (0.367)	0.090 (0.250)	0.447 (−0.068)	0.172 (0.285)
High greenery	0.059 (0.604)	0.974 (−0.018)	0.048 (0.436)	0.757 (0.063)	0.772 (0.041)	0.899 (−0.011)	0.260 (0.229)
Winter	0.859 (0.057)	0.640 (−0.265)	0.003 (0.669)	0.966 (0.009)	0.881 (−0.022)	0.411 (0.072)	0.569 (0.116)
Gender (Male)	0.976 (−0.007)	0.014(−0.961)	0.408 (-0.125)	0.661 (−0.063)	0.993 (0.001)	0.624 (0.030)	0.910 (−0.016)
Age (25–44)	0.046 (−0.621)	0.084 (0.919)	0.025 (−0.469)	0.517 (−0.125)	0.530 (0.085)	0.836 (0.017)	0.690 (−0.076)
Age (45–65)	0.005 (−1.147)	0.003 (2.087)	0.005 (−0.766)	0.705 (0.098)	0.294 (0.191)	0.075 (−0.196)	0.709 (0.096)
Stressful day	0.145 (0.089)	0.400 (−0.087)	0.506 (−0.027)	0.945 (0.003)	0.957 (0.001)	0.258 (−0.019)	0.186 (0.051)
Physical health	0.958 (−0.008)	0.170 (0.367)	0.921 (−0.010)	0.074 (0.171)	0.449 (−0.050)	0.293 (0.042)	0.081 (−0.165)
Mental health	0.256 (−0.192)	0.193 (−0.378)	1.000 (0.000)	0.005 (0.300)	0.842 (0.015)	0.070 (0.081)	0.199 (−0.133)
Nature connectedness	0.030 (0.226)	0.221 (−0.214)	0.548 (−0.041)	0.081 (0.117)	0.600 (0.024)	0.479 (0.020)	0.118 (0.103)
Cycling frequency	0.491 (0.073)	0.786 (−0.049)	0.399 (0.059)	0.998 (0.000)	0.882 (−0.007)	0.069 (−0.051)	0.375 (0.058)

Analysis of variance partitions (Supplementary 3. Variance partition plots for the regression models) of these regression models revealed that the proportion of the total variance explained by these models was modest, ranging from 3% (PANAS-POS change) to 17% (PANAS-NEG change). In practice, for instance, 5% of observed variance in pupil diameter was associated with environment type, while this was only 1% for the blink rate. Age explained the largest fraction of variance for mean blink rates and pupil diameter, SCL change and PANAS-NEG change, mental health for the SVS and ROS change (3% and 7% respectively), and environment type for the PANAS-POS change (~ 2%).

### Temporal effects on physiological measures

**Fig. 4:**
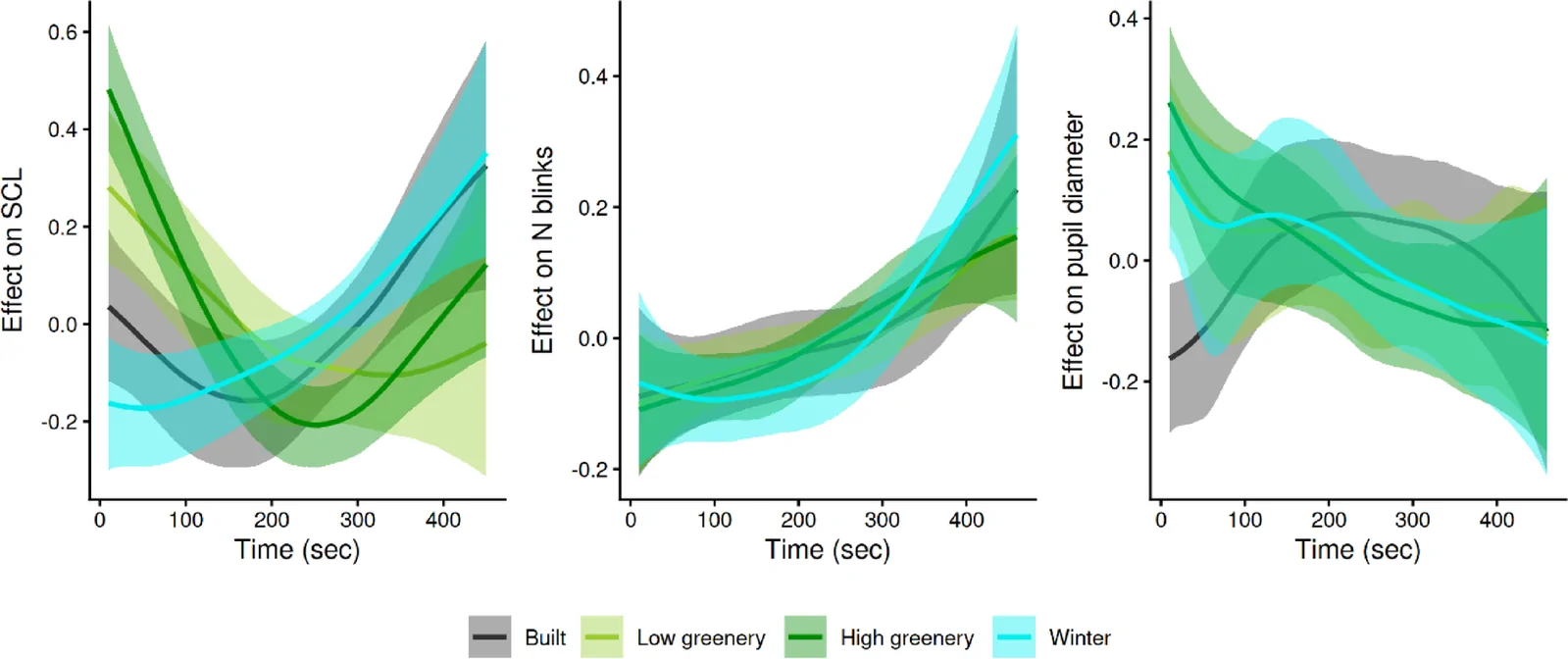
Estimated marginal effects of the four environments in time (sec) on mean baseline-adjusted Skin Conductance Level (SCL), blink rate (N blinks) and pupil diameter (dm), according to the three Bayesian temporal models during the Recovery period.

**Fig. 5:**
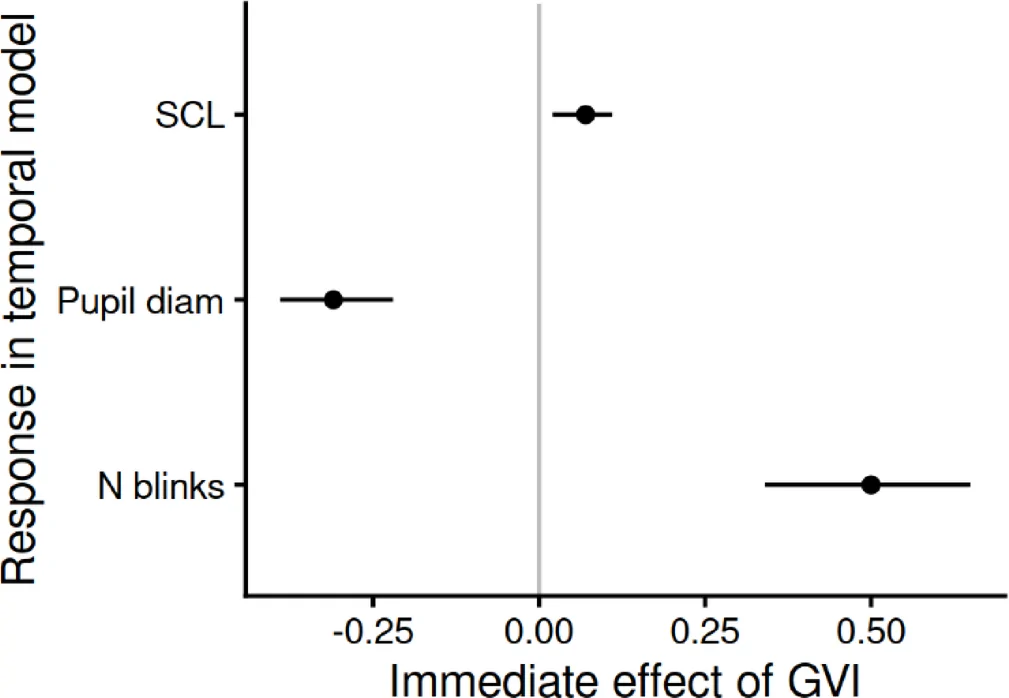
Estimated marginal effects of GVI from the three temporal models (baseline-adjusted Skin Conductance Level (SCL), pupil diameter and number of blinks).

We further assessed the temporal dynamics of physiological outcomes (baseline-adjusted SCL, blink rate and pupil dm) within the 8-minute cycling exposure using a Bayesian temporally-explicit regression model, with the individual GVI exposure values as a covariate. Figures 4 and 5 show the estimated marginal effects of the environment type (in time) and GVI under the three fitted models. We found clear effects of GVI on the physiological measurements. GVI had a significant positive effect on SCL and number of blinks, and a clearly negative effect on pupil diameter (Fig. 5). When looking at the temporal effects of the VR environments on the measured physiological responses of cyclists, we found that in the three summer conditions (built, low and high greenery) the SCL levels decreased after the induced stress with greatest reduction effect around 3–5 min of exposure, after which SCL in all three environments started to increase (Fig. 4). Notably, however, the decrease in SCL was estimated to be largest in the high greenery environment and lowest in the built environment, suggesting a different effect of the two environments during cycling. In addition, in the winter condition, the overall reduction in SCL in the early recovery phase could not be observed. Instead, the effect of winter environment on SCL level was consistently increasing in time, suggesting a cumulative arousal. Regarding the effect sizes, we note that the individual level variance in adjusted SCL varied mostly between 0.037 and 1.543 (5% and 95% empirical quantiles), so the estimated effects appear relevant in size.

When analysing the temporal effects of the VR environments on blink rates, no clear differences were found between the environments with a generally increasing positive effect towards the end of the cycling Recovery period. On the other hand, individual pupil diameter decreased with time in all vegetation environments. However, a different pattern was observed in the built environment, in which the pupil diameter was inferred to first increase in time and then decreased only towards the end of the recovery period. In all temporally-explicit models, high degree of autocorrelation between the consecutive measurements from the same individuals was found (0.99 for SCL, 0.93 for pupil diameter, 0.96 for blinking).

## Discussion

### Street greenery improves mood, but restoration effects were mixed

In this VR experimental study, we tested the effects of different street greening designs and seasonal conditions on the restoration, affective responses and subjective vitality of cyclists. Our findings partially support the pre-defined hypotheses. First, we hypothesised that street greenery increases psycho-physiological restoration and improves mood and subjective vitality. We found significant effects only for some measures. Cyclists’ exposure to both greenery environments (low and high greenery) significantly decreased negative affect (PANAS-NEG) compared to pre-experimental measures, which contributes to previous findings^52,53^. However, although participants reported increase in positive affect (PANAS-POS) and subjective vitality (SVS) after cycling in the two greenery environments, these changes were not statistically significant. This is not completely in accordance with previous studies^53,54^, which can be potentially attributed to the difference in between- or cross-over experimental designs. It should be also considered that while our experiment had four conditions, these studies have compared two very distinctive VR environments (control vs. nature; urban vs. nature), which may have contributed to the stronger difference in positive effect.

Further, in line with our first and second hypothesis, the high greenery environment, which had the highest level and diversity of natural auditory-visual elements, lowered individual SCL levels the most after the induced stress. This is in line with stress reduction theory^43^ and other outdoor^55^ and laboratory experiments^56,57^. This result also confirms previous findings that stress reduction is more effective within a combined auditory-visual stimuli (e.g. bird songs)^29,54,56,58^, although here we did not test for separate effects or interactions of these modalities. Recent studies have highlighted that soundscape attributes can have important positive non-linear effects on cycling behaviour, especially when there is audio-visual consistency between natural sounds and visual openness^5,13,59^. This is particularly relevant for future VR laboratory experiments, where researchers can control for sound sources, intensity and frequency of exposure^56^.

Furthermore, similar to previous experiments conducted while sitting^55,56,60^, the greatest stress reduction was reached around 4–5 min, showing momentary recovery potential of natural environments even under rapid exposure during a cycling commute. This finding implies that urban planning should ensure 1–2 km continuous vegetation sections in cycling corridors in order for stress reduction effects to occur. Since many cycling trips are short distance and for non-commute purposes, such recommendations can be expanded beyond commuting corridors to making neighbourhoods and school routes combine both greenery and accessibility^61,62^. In addition, previous research has shown that associations between environmental characteristics and pleasantness during commute and non-commute physical activity are highly contextual and spatially non-stationary^6,63^, which highlights the need for locally designed greenery interventions. We warrant for future research to further test the cumulative effects of continuous street vegetation exposure, as well as possible effects of repetitive exposure or distance decay in everyday travel.

The effects of street greenery on the eye-tracking measures were also confirmative to our hypotheses. Higher GVI increased blink rates and decreased pupil dilation in the Recovery period, which suggests reduction in directed attention and stimuli engagement. In addition, pupil dilation continuously decreased with time in all greenery conditions, but it took longer time for participants in the built environment group to reach such decrease only towards the end of the experiment. This is consistent with the soft fascination dimension of ART theory^44^, which proposes that natural environments can evoke effortless attention allowing recovery of directed attention and the opportunity for reflection.

However, self-reported restoration (ROS measures) increased after exposure to all VR cycling environments regardless of the condition, and similarly blink rates increased with time in all environments, suggesting accumulative effect on reduced directed attention. Also surprisingly, the built environment, which had no vegetation or birdsongs, had a perceived restorative effect and slightly reduced physiological arousal after the stress period. This suggests that cycling in VR as a form of exercise can relieve stress and contribute to cognitive restoration^64^. The lack of traffic, people or animals in our VR designs may have also contributed to this effect. Similarly, other studies have showed that virtual reality itself can have positive restorative impacts regardless of the condition. For example^65^, found a recovery effect for individuals with mild-to-moderate anxiety and depression in both urban and natural VR conditions, and the authors suggested that having a quiet urban environment without people can have a healing effect. In addition^66^ and^53^ showed that exposure to any VR condition reduced heart rate. Future research could include the role of sense of presence (i.e. the sense of being there) in VR and how it affects emotional responses and restoration mechanisms^67^. The strong sense of presence in VR environments may contribute to the feeling of “being away” which is another important aspect of Attention Restoration Theory^44,68^.

Finally, there was a difference in stress reduction in the summer compared to winter VR environments which supports our third hypothesis. Participants who cycled in the winter condition did not show reduction from the stressor and instead had a consistently increasing SCL response pattern, indicating a possible unique underlying mechanism. However, other results were not statistically significant, which corroborates the inconclusive findings in previous research. For example, results from a general population study in the Netherlands did not support the widespread belief that seasons influence mood to a great extent^69^. Previous research by^46^ also showed that exposure to virtual nature generally induce similar psycho-physiological responses regardless of season, except for stronger reduction in feelings of fatigue during autumn and winter. A limitation of this study is that we included only two seasons (summer and winter) to balance the resources and time needed for development, and reduce the complexity of the experimental design. Further research across all seasons is needed since studies assessing seasonal variation in benefits of greenery during cycling are still scarce.

### Higher reactivity to natural elements in VR

Our findings showed that higher street greenery exposure (GVI) had a statistically significant positive effect on SCL values, which was a surprising result and contrary to the first hypothesis. In addition, higher nature connectedness of participants was also associated with increased SCL values. Despite previous evidence that biophilic design can reduce stress and anxiety^60^, contrasting results have been found by other laboratory studies indicating increase in skin conductance during exposure to simulated nature^52^ or natural (wooden) elements in an office setting^70^. Such arousal may reflect that the natural visual and auditory stimuli in virtual conditions may have an amplifying effect. In addition, in our study, none of the VR conditions were threatening or included any living beings such as people or animals, except the birdsongs that could be heard in the three greenery conditions. This may have caused participants, especially those that feel more connected to nature, to be more reactive to the vegetation elements and accompanying birdsongs as they are the only living elements in the otherwise rather static virtual world. Overall, our scenarios allowed testing greenery and seasonality effects in safe conditions, but this naturally brings challenges with generalising the findings into real-life urban travel environments.

Generally, SCL indicates only arousal of the sympathetic nervous system without showing valence i.e. how positive or negative an experience feels, which is the second key dimension in affective experience^71^. Although humans need to recover from both positive and negative arousal, moderate level of pleasant arousal could also increase alertness, the quality of sensory stimuli (more information made available per unit time) and increase short-term and long-term memory^72,73^. ^52^also showed that increased physiological arousal during virtual nature exposure was associated with the increase or maintenance of positive affective states. In this study, participants reported slight non-significant increase in positive affect scores after cycling in the greenery conditions, which suggests that higher arousal occurred at the same time as positive emotions were maintained. Although this requires further investigation, it generally highlights the important role of pleasantness on psycho-physiological restoration effects in green areas^26,55^ and during active mobility^6,9^.

### Differences between the greenery environments were hard to detect in VR

Generally, differences in psycho-physiological responses to cycling especially in the high and low greenery conditions were not that obvious, and therefore, we could not confirm the second hypothesis. One explanation for lack of significant results could be that our computer-generated environments and greenery levels (low greenery µGVI = 0.221 and high greenery µGVI = 0.282) were simply not different enough to capture subtle or important elements that may shape human experience and perceptions. Future research can include self-reported ratings of visual greenery, complexity or level of naturalness to better account for perceptual differences between the experimental conditions. Similar lack of significant effects between greenery conditions were reported in previous studies. For example^74^, found that VR environments with high level of greenery (50%) reduced both physiological and affective stress for pregnant women compared to a non-green environment (0%), but the same effects were not found for the moderate level (12%). Also, interestingly and contrary to our hypothesis, the low greenery condition was associated with highest mean increase in self-reported restoration and highest blink rate, which suggests stronger effects in cognitive restoration. Past studies have also shown that a lower biodiversity VR environment can more effectively lower anxiety and stress compared to an urban and higher biodiversity VR environment^58^.

In our study, the cycling may also play an important role since previous research has demonstrated trade-offs between level of greenery and audio-visual complexity during active movement^13,75^. The authors suggested that landscapes that contain too much diversity in size, form, texture, and spatial characteristics may require additional directed attention and arousal, and therefore weaken restorative effects while on the move^75^. Also too dense vegetation may reduce the visual openness and perceived safety of cyclists^76^. Our study is one of the very few experiments on nature exposure while cycling in VR^35^and further research is needed to disentangle the effects of emotion, restoration and physical activity during active travel. Although we used a cycling baseline in a neutral VR environment to control for the effects of physical exercise, still we could not fully account for the confounding effects of movement. This is due to two important limitations in our experimental design: the lack of baseline eye-tracking data and repeated measures of psychological outcomes after each experimental phase. For example, blink rates are also shown to increase with increase in fatigue^77^. Therefore, it is advisable to collect an individual baseline for the eye-tracking data during cycling in a neutral VR condition to allow for distinguishing eye physiology as part of physical activity only compared to physical activity under a treatment condition. This can help create a moving baseline accounting for natural drift in the blink data. To reduce physical load, baseline measures should be kept short (up to 3 min) and brief rest periods can be introduced between experimental phases. Future studies can also include visual information processing aspects such contrast, spatial frequency, colour^78^, as well as visual enclosure, sky openness or depth of field^5,13,76,79^.

Finally, we acknowledge that our sample was biased towards younger and highly educated participants, which limits the generalisability of the findings to broader socio-demographic populations. In line with recommendations from the wider research community, future studies should prioritise targeting different socio-cultural groups in order to move beyond the dominant Western, Educated, Industrialized, Rich, and Democratic (WEIRD) sample in psychological and behaviour research^80,81^.

## Methods

This study received an ethical approval statement by the Research Ethics Committee in the Humanities and Social and Behavioural Sciences at the University of Helsinki (48/2024), stating that all methods were carried out in accordance the relevant guidelines and regulations for research in the humanities and social and behavioural sciences issued by the Finnish Advisory Board on Research Integrity. Power analyses were conducted prior to the experiments to ensure sufficient statistical power using the “simr” package (v.1.0.9)^82^ in the R software. The power analyses were based on self-reported restoration scores and indicated that *N* = 140 represents the smallest sample size where the lower limit of the 95% confidence interval of the power curve exceeds 0.80. The study was pre-registered in OSF in February, 2025 (https://doi.org/10.17605/OSF.IO/XA4CD).

### Participants

Finally, a total of 151 participants took part in VR experiments between February - May 2025. The participants were recruited as a convenience sample using university emails, posters and social media. The inclusion criteria were that study subjects are 18–65 years-old healthy individuals, while no prior VR or cycling experience was required. Mental and physical health and use of anticholinergic medications was self-reported in the survey. Participants were instructed to avoid alcohol and caffeinated drinks consumption at least two hours before the experiment.

After accounting for data loss due technical issues and excluding individuals for which the stress test did not work, the final sample included a total of 138 participants that were a mixture of university students and staff, as well as the general public. The sample included 58.7% female/38.4% male, mostly 25–44 years old (67.4%) individuals with generally high education level (79.8% with Bachelor degree or higher). The participants reported good mental (mean = 3.78) and physical (mean = 3.88) health, and not very stressful day before the experiments (mean = 3.97, 10-point scale). In total 71.9% of participants stated to have experienced Virtual Reality before. Participants also reported generally no cyber sickness symptoms during the experiment (mean = 1.32) based on the four-point Likert scale (1 = none; 4 = severe) of the Simulator Sickness Questionnaire^83^. Chi-squared tests indicated no statistically significant differences in background variables (age, gender, education, stressful day, physical health, mental health, cycling frequency and nature connectedness) between study subjects in the different groups, indicating successful randomisation.

### Study design and experimental procedure

The experiment used a between-subjects design and each participant was blindly randomised to only one of four environmental VR conditions: one built and three greenery scenarios. We opted for a between-subjects design for two main reasons: to avoid carry-over effects of stressors and the VR environments within subjects, and to reduce the time of wearing the headset, which can cause discomfort or cybersickness due to prolonged presence in VR^84^.

**Fig. 6:**
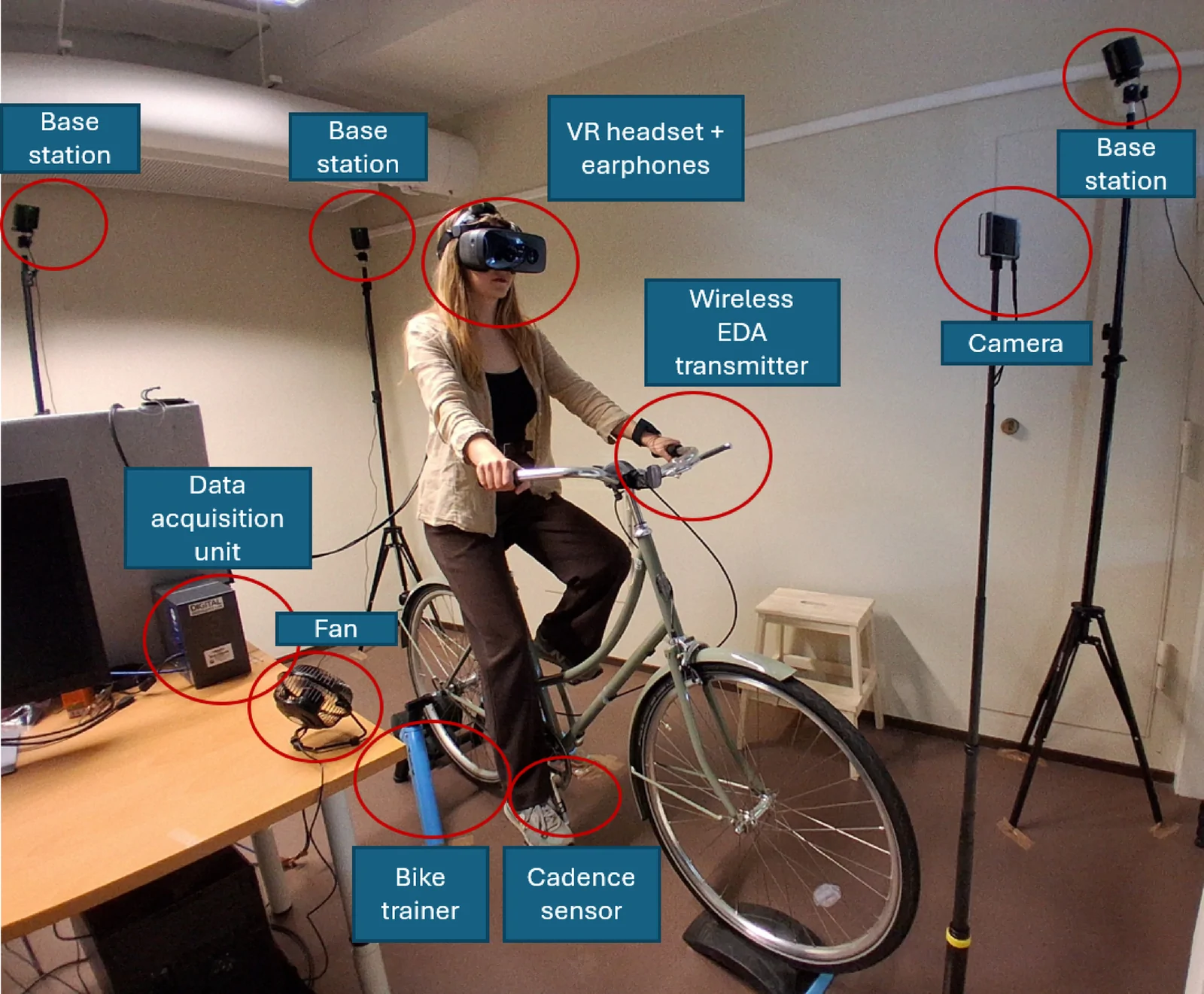
Experimental set up and hardware components.

**Fig. 7:**
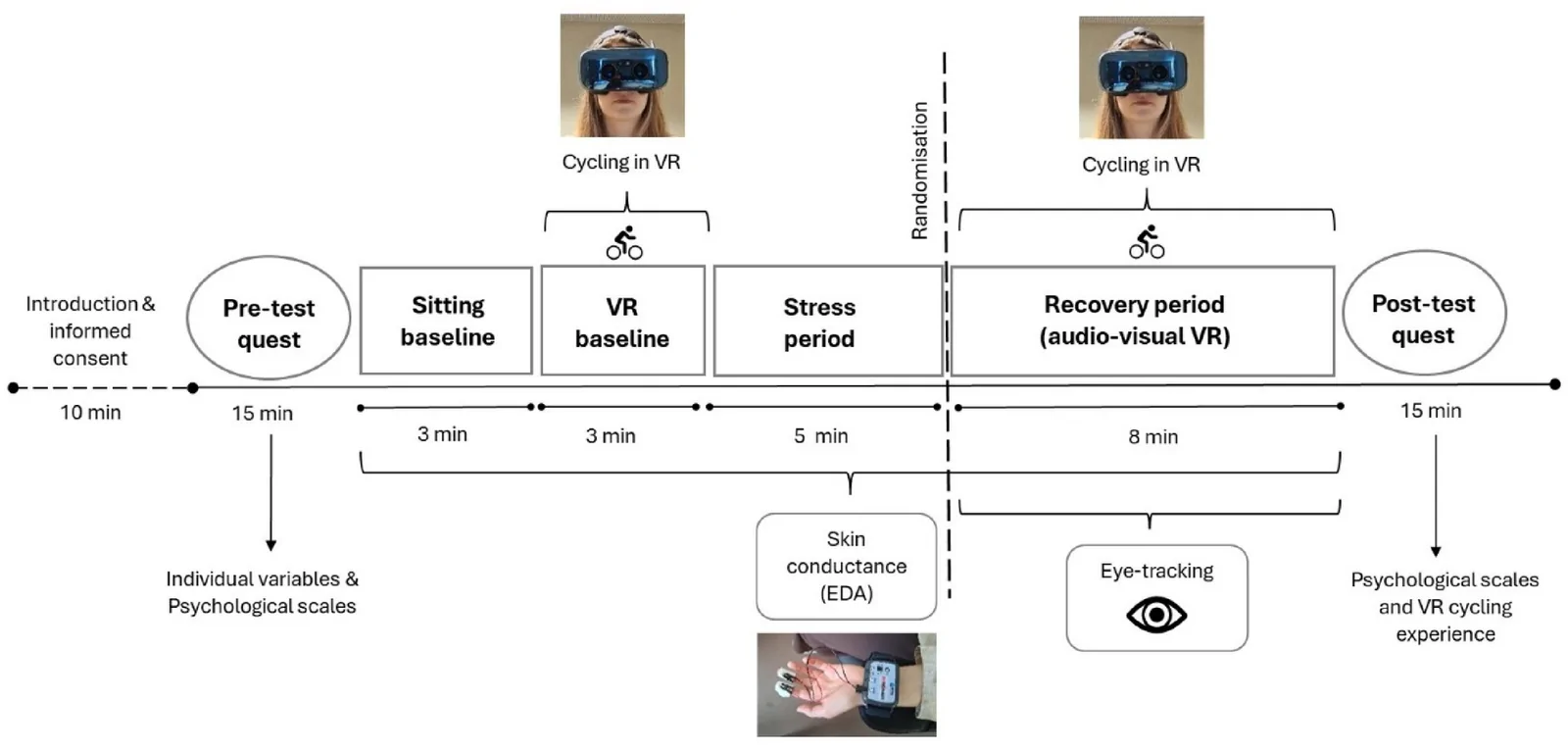
Different phases of the VR experiment.

The experiments were carried out in a laboratory setting at the university premises. The temperature, humidity and light conditions in the room were kept consistent. The experimental set up consisted of a bicycle with an attached Garmin Tacx bike trainer, a Varjo XR-3 MR headset, wireless earphones, and wireless BioNomadix transmitter bracelet measuring Electrodermal Activity (EDA) through skin conductance (see Fig. 6 for demonstration by a member of the research team). Skin Conductance Levels (SCL) was acquired continuously at a 2000 Hz rate using two dry electrodes on the middle and index fingers. A small fan for simulating breeze during cycling was used to increase the multisensory experience. Detailed description of the experimental set up, hardware and software components, codes and data collection protocols can be found in https://github.com/DigitalGeographyLab/GREENTRAVELVR.git and^85^. Informed Consent for publication of images were obtained (Figs. 6 and 7).

The experiment including several phases and it took approximately 1–1,5 h (Fig. 7). Prior to the experiment, participants were sent general instructions, overview of the research study and a Data Privacy Statement. Upon arrival, participants were welcomed and shown a short introduction video of the different phases and devices used. Then, they signed the informed consent form and filled in a pre-questionnaire on their background and current perceived restoration and affective state. Then, two baselines were taken: a three-minute baseline for measuring SCL during rest while sitting on a chair, and a three-minute cycling baseline in VR, during which the participants cycled in a neutral environment with no sound. The VR cycling baseline served two purposes: as a habitualisation period to help participants get used to the cycling experience and the VR effects, and to measure a skin conductance baseline level during physical movement (cycling). After the VR baseline, participants were stressed for five minutes using a mental arithmetic stress task by subtracting backwards 17 from 1000 and having five sec for each response. Participants’ responses were evaluated as either “correct”, “false” or “too late” (if later than 5 s) and incorrect responses led to participants starting from the beginning. The effect of stress, measured as the difference in the mean SCL levels between the stress period and sitting baseline, was significant (p = < 0.001) and positive, indicating that the mental arithmetic test induced stress successfully. There were no statistically significant differences between the groups in the effect of stress.

After the stress test, participants cycled in one of the four randomised VR recovery conditions. Participants could cycle only in a straight line but could turn their head and look around. They were instructed to cycle at a leisurely speed, and the speed was topped at 15 km/h to reduce the physiological effects of rigorous exercise. To simulate an everyday active behaviour, participants were asked to imagine that “it is an early morning and they are cycling to work, but on the way to work they need to pick up some food from the supermarket”. Their task was to reach the supermarket which was located at the end of the road. After the Recovery period (8 min), participants went back to sitting on a chair and filled a post-questionnaire including the same psychological scales, as well as their overall experience and cybersickness symptoms. All participants were given a 20-euro grocery store voucher or two movie tickets as compensation.

### Virtual environments

The Recovery period included cycling in a VR environment with a 1.5 km long straight street, which represents a typical distance for active mobility^61,86^. The VR environments were designed though an iterative process in a transdisciplinary team including VR technicians, urban planners and researchers with expertise in transportation, environmental psychology, health, ecology, and landscape architecture. The environments were generated in Unreal Game Engine (Epic Games) having built infrastructure and street dimensions with real-life proportions. To avoid place attachment and familiarity bias in responses, the environments were created to simulate the feeling of cycling in a city without being in a recognisable place. To increase ecological validity, vegetation assets and birdsongs were chosen to be typical for Nordic countries. High visual quality and multisensory experiences (auditory, wind through the fan) were used to increase sense of presence in the virtual world^41^. Since the experiments were conducted in Helsinki, local 3D assets (e.g. bus stops, traffic signs) were also used to increase contextual realism. In addition, no people or moving cars were present in the VR environments to provide feeling of safety and avoid confounding effects on physiological arousal.

We developed four VR environments representing different street greening designs and seasonal conditions: built, low level of greenery, high level of greenery and winter (Fig. 1, example videos also available in the GitHub dataset https://github.com/DigitalGeographyLab/GREENTRAVELVR.git). All environments had identical built infrastructure and distant ambient traffic noise, but vegetation and birdsongs were varied to respond to the diversity and level of naturalness. The built, low and high greenery environment also had the same light and seasonal conditions. The environments included the following elements:

1. *Built*: control urban environment with very limited number of trees and vegetation; ambient traffic sound (continuous); summer sky and lighting conditions.
2. *Low greenery*: low level and diversity of greenery with monotonous tree-lined vegetation (trees and grass), limited number of bushes; in addition to the traffic sound, four times birdsong of Common Blackbird (*Turdus merula*) was played as spatial audio; summer sky and lighting conditions.
3. *High greenery*: higher quantity and more varied vegetation with structural diversity, more vegetation layers including bushes and seasonal pollinator flowers; green walls and other vertical vegetation; in addition to the traffic sound, eight times birdsong of Common Blackbird (*Turdus merula*), Chaffinch (*Fringilla coelebs*), or Great tit (*Parus major*) was played as spatial audio; summer sky and lighting conditions.
4. *Winter*: a multi-feature winter composite condition as a seasonal variation of the high greenery environment, same trees and bushes without leaves; snow piles randomly located on the ground; in addition to the traffic sound, one time a birdsong of House Sparrow (*Passer domesticus*) was played as spatial audio; winter overcast sky and low light condition.

### Measures and data processing

#### Physiological measures

Skin conductance data was processed in AcqKnowledge programmme (BIOPAC Systems, Inc.), which was synchronised in real-time with the VR environments in Unreal Engine. We used tonic activity (skin conductance level) as outcome variable since it reflects progressive changes in galvanic skin response over time^87^. SCL data was first resampled to 200 Hz with a digital low pass filter at 1 hz. We then conducted visual investigation of the signal and correction of artefacts. Naturally, there is a high degree of variability across individuals in SCL responses. Therefore, we used an established approach of determining the relative changes of each participant average SCL during the experiment. SCLs were averaged every 10 s and adjusted to baseline measures for each individual according to the two types of physical state: sitting or cycling. Stress period SCL values (while sitting on the bike) were adjusted to the mean value of the last two minutes of the sitting baseline (ΔSCL_stress_ = SCL_stress_− SCL_sit_baseline_). Recovery period (cycling in VR) was adjusted to the mean value of the last two minutes of the VR cycling baseline period (ΔSCL_Recovery_ = SCL_VR_Recovery_− SCL_VR_baseline_). Adjusting recovery values to cycling in the neutral environment also allows for distinguishing the effect of the physical movement.

Pupil diameter and blink rates were collected with the built-in eye tracking features of the Varjo XR-3 headset. For the pupil diameter, we used the average diameter of both pupils. For blink rates, we used data loss on variables *right_status* and *left_status* in the eye-tracking system as a proxy for blinks^88^. Closures of the eye less than 30 ms and longer than 400 ms were ignored, while neighbouring blinks with gaps shorter than 100 ms were combined^88–90^. Lastly, the eye-tracking data was averaged for the 10 s intervals similarly to the SCL measures.

#### Psychological measures

Different psychological scales were used to measure participants’ perceived restoration, affective state and subjective vitality, which have been widely adopted in previous similar studies^28,29,55,70^. All scales were used before (pre-questionnaire) and after the experiment (post-questionnaire) to identify change in mental states.

The Restoration Outcome Scale (ROS)^91^ was used to measure self-reported restoration including six items (“I feel calm”, “I can concentrate well and feel alert”, “I have enthusiasm and energy for my everyday routines”, “I feel restored and relaxed”, “I can forget everyday worries”, “My thoughts are clear”). Self-reported emotional state was measured using the international Positive and Negative Affect Schedule short form (I-PANAS-SF) (Thompson, 2007), which is found to be a concise and reliable shorter version of PANAS^92^ suitable for various contexts. The scale includes five positive (Alert, Inspired, Determined, Attentive, Active) and five negative items (Upset, Hostile, Ashamed, Nervous, Afraid). To measure self-reported feeling of energy, the subjective vitality scale (SVS)^93,94^ was used including four of the original six items (“I feel alive and vital,” “I have energy and spirit,” “I look forward to each new day,” and “I don’t feel very energetic” as reversed item). The four items were selected to respond to local language nuances following similar approach to previous studies in the Finnish context^28,95^. ROS and SVS were rated on a seven-point Likert scale (1 = Not at all, 7 = Completely), and PANAS was rated on a five-point Likert Scale (1 = Very slightly or not at all, 5 = Extremely). All item scores were averaged and differences in pre and post responses were calculated for each participant.

#### Covariates

Several individual background variables were collected in the experiment including age, gender, education, self-reported overall physical and mental health (single-item WHO 5-point Likert scale; 1 = very bad, 5 = very good)^96^; how stressful was participants’ day before the experiment (10-point Likert scale, 0 = not stressful at all, 10 = extremely stressful), if they have used virtual reality before (yes/no), subjective connectedness with nature using the Inclusion of Nature in Self (INS) scale^97^, and participants cycling frequency (5-point Likert scale; 1 = less than once a month; 5 = 5–7 days/week).

#### Individual exposure to greenery

The Green View Index (GVI)^98^, the amount of visible greenery exposure of participants, was calculated by segmenting the participants headset video recordings at one second intervals. We used a pretrained ADE20K Dinat-L trained variant of the OneFormer Semantic segmentation model from the Hugging Face platform^99–101^. GVI was then calculated by assessing the share of the classes *tree*, *grass*, *plant*,* flower* from the total share of pixels and then, similar to the other measures, averaged for each 10 s interval.

### Statistical analysis

First, we tested whether the environment type affects changes in psychological outcomes (ROS, PANAS-POS, PANAS-NEG, SVS) and the mean physiological outcomes (baseline-adjusted SCL, Pupil diam and N blinks). We fitted separate gaussian regression models to all these outcomes, using the environment type together with individual background variables (gender, age, stressful day, self-reported physical and mental health, nature connectedness and cycling frequency) as covariates. Here environment type, gender, and age groups were treated as categorical covariates, for which an effect was estimated for every factor level besides the baseline, while for the other covariates the effect was assumed to be linear.

We further tested the effect of the environment and the visible street greenery level (GVI) on the instantaneous physiological responses of each participant within the 8-min recovery period. We modelled the physiological response (below SCL) of participant *i* at time *t* in environment E(i) with an autoregressive regression model as follows:

$$SCL_{{i,t}} \sim Normal\left( {\mu _{{i,t}} ,\sigma _{i} } \right)$$

$$\:{\mu\:}_{i,t}\:=\varphi\:{\mu\:}_{i,t-1}+\beta\:GV{I}_{i}\left(t\right)+{s}_{E\left(i\right)}\left(t\right)$$

Here $\:GV{I}_{i}\left(t\right)$ stand for the measured Green View Index of the participant *i* at time *t* (and 10 s before), $\:{s}_{E\left(i\right)}\left(t\right)$ stands for the effect of the environment E(i) on the outcome as a function of time, and first term accounts for the autocorrelation in physiological measurements within individuals, where parameter ψ describes the degree of autocorrelation between the consecutive measurements from the same individual. The effects of the environments $\:{s}_{E\left(i\right)}\left(t\right)$, are assumed to be smooth non-linear functions in time, and are estimated from data. In addition, we assumed individual specific variation in SCL values, parameterized with $\:{\sigma\:}_{i}$, around their expected value. We assumed that this variation is constant throughout the experiment and unique for each individual, but assumed the $\:{\sigma\:}_{i}$‘s come from a shared population distribution.

Similar model structure was used to model pupil diameter and number of blinks during the Recovery period. For the SCL and pupil diameter measurements we assumed a Gaussian sampling distribution, while for the Number of blinks we assumed the outcomes to have a Poisson distribution. We did not model the individual-specific variances in the measurement outcomes for the number of blinks.

Thus, our target of inference are the following parameters ψ, β,$\:{s}_{E\left(i\right)}\left(t\right)$, where the smooth effects of the environments are estimated for each of the four categories: built, low greenery, high greenery and winter. With $\:{\sigma\:}_{i}$‘s we adopted hierarchical modelling approach, in which we learn the population distribution of σ_i’s from data simultaneously with the individual $\:{\sigma\:}_{i}$‘s. We did Bayesian posterior inference on the model parameters, implementing the model fitting with MCMC sampling using brms-package (v.3) in R^102^, using default priors of the software for all the parameters. For each model we ran 4 MCMC chains, each with 5000 iterations, for which 2500 iterations were considered as warmup. This resulted in appropriate convergence diagnostics for all the models, e.g. Rhats being almost equal to one for all the parameters. Trace plots also indicated appropriate convergence of the chains.

## Supplementary Information

Below is the link to the electronic supplementary material.

Supplementary Material 1

## Data Availability

The virtual environments source codes, documentation and data collection protocols are available from GitHub repository [https://github.com/DigitalGeographyLab/GREENTRAVELVR.git].
