# Supplementary material for: Effects of street greenery and seasonality on health and wellbeing of cyclists: a Virtual Reality experiment
Source: Sci Rep. 2026 Jul 29;16:34910. doi: 10.1038/s41598-026-64499-4 (PMC13713577; doi:10.1038/s41598-026-64499-4)
Supplement: Supplementary file 1 [file 41598_2026_64499_MOESM1_ESM.pdf]

Supplementary material

Environment	Measure	N	Mean Pre	Mean Post	Mean Diff	t	p	Cohen's d	p (bonf)
Built	ROS	31	3.597	5.156	1.559	9.376	0.0000	1.684	0.0000
LG	ROS	34	3.574	5.143	1.569	10.955	0.0000	1.879	0.0000
HG	ROS	38	3.584	4.891	1.307	9.836	0.0000	1.596	0.0000
Winter	ROS	35	3.548	4.775	1.226	7.801	0.0000	1.319	0.0000
Built	SVS	31	5.532	5.476	-0.056	-0.368	0.7152	-0.066	1.0000
LG	SVS	34	5.088	5.316	0.228	1.659	0.1065	0.285	1.0000
HG	SVS	37	4.851	5.124	0.272	2.417	0.0209	0.397	0.3336
Winter	SVS	35	5.057	5.186	0.129	0.871	0.3898	0.147	1.0000
Built	PANAS-POS	31	3.661	3.631	-0.031	-0.308	0.7599	-0.055	1.0000
LG	PANAS-POS	34	3.400	3.565	0.165	1.815	0.0786	0.311	1.0000
HG	PANAS-POS	38	3.200	3.237	0.037	0.404	0.6888	0.065	1.0000
Winter	PANAS-POS	35	3.326	3.254	-0.071	-0.813	0.4220	-0.137	1.0000
Built	PANAS-NEG	31	1.326	1.226	-0.100	-1.419	0.1663	-0.255	1.0000
LG	PANAS-NEG	34	1.371	1.182	-0.188	-4.217	0.0002	-0.723	0.0029
HG	PANAS-NEG	38	1.411	1.253	-0.158	-3.224	0.0026	-0.523	0.0423
Winter	PANASNEG	35	1.411	1.360	-0.051	-0.704	0.4861	-0.119	1.0000

Table 1. Within environment change of mean values of the psychological measures: Paired t-test with Bonferroni correlation on all 16 tests; Cohen's d for effect size. LG=Low Greenery, HG = High Greenery

2. Results of the regression models

We fitted linear regression models to the following response variables using the same set of covariates: environment (environment_ID), gender, age, stressful day (stress_day), physical health, mental health, nature connectedness (Self_nature) and cycling frequency (Cycling_Freq). Here environment, gender and age were categorical values and thus fitted as factors. The environment (built), gender (female) and age (18-24) were treated as baseline factor levels and the effects of other factor levels were estimated relative to those. Below the regression model results are reported for each response variable: linear effect, standard error, t-value, significance level, the lower boundary and the upper boundary of the 95% confidence interval.

ROS_change: change in pre-post mean scores of the Restoration Outcome Scale (Korpela et al., 2008)

```
                           Estimate Std. Error t value Pr(>|t|)  2.5 % 97.5 %
(Intercept)                  -0.954      0.608  -1.569    0.119 -2.145  0.238
Environment_IDLow greenery    0.367      0.210   1.748    0.083 -0.044  0.777
Environment_IDHigh greenery   0.063      0.202   0.310    0.757 -0.334  0.460
Environment_IDWinter          0.009      0.206   0.042    0.966 -0.394  0.412
as.factor(Gender_Male)       -0.063      0.144  -0.440    0.661 -0.346  0.219
as.factor(Age_25_44)         -0.125      0.192  -0.650    0.517 -0.502  0.252
as.factor(Age_45_65)          0.098      0.259   0.379    0.705 -0.409  0.605
Stress_day                    0.003      0.039   0.069    0.945 -0.073  0.079
Physical_health               0.171      0.095   1.803    0.074 -0.015  0.356
Mental_health                 0.300      0.104   2.881    0.005  0.096  0.505
```

Self_nature	0.117	0.066	1.761	0.081	-0.013	0.247
CyclingFreq	0.000	0.065	0.002	0.998	-0.128	0.128

PANAS_POS_change: change in pre-post mean scores of positive dimension of the international Positive and Negative Affect Schedule short form (Thompson, 2007),

	Estimate	Std. Error	t value	Pr(>|t|)	2.5 %	97.5 %
(Intercept)	-0.101	0.426	-0.236	0.814	-0.935	0.734
Environment_IDLow greenery	0.250	0.147	1.706	0.090	-0.037	0.538
Environment_IDHigh greenery	0.041	0.142	0.290	0.772	-0.237	0.319
Environment_IDWinter	-0.022	0.144	-0.150	0.881	-0.304	0.261
as.factor(Gender_Male)	0.001	0.101	0.009	0.993	-0.197	0.199
as.factor(Age_25_44)	0.085	0.135	0.630	0.530	-0.179	0.349
as.factor(Age_45_65)	0.191	0.181	1.054	0.294	-0.164	0.546
Stress_day	0.001	0.027	0.054	0.957	-0.052	0.055
Physical_health	-0.050	0.066	-0.760	0.449	-0.180	0.080
Mental_health	0.015	0.073	0.199	0.842	-0.129	0.158
Self_nature	0.024	0.047	0.526	0.600	-0.067	0.116
CyclingFreq	-0.007	0.046	-0.149	0.882	-0.097	0.083

PANAS_NEG_change: change in pre-post mean scores of negative dimension of the international Positive and Negative Affect Schedule short form (Thompson, 2007)

	Estimate	Std. Error	t value	Pr(>|t|)	2.5 %	97.5 %
(Intercept)	-0.455	0.257	-1.769	0.079	-0.959	0.049
Environment_IDLow greenery	-0.068	0.089	-0.762	0.447	-0.242	0.106
Environment_IDHigh greenery	-0.011	0.086	-0.128	0.899	-0.179	0.157
Environment_IDWinter	0.072	0.087	0.825	0.411	-0.099	0.243
as.factor(Gender_Male)	0.030	0.061	0.492	0.624	-0.090	0.149
as.factor(Age_25_44)	0.017	0.081	0.207	0.836	-0.143	0.176
as.factor(Age_45_65)	-0.196	0.110	-1.793	0.075	-0.411	0.018
Stress_day	-0.019	0.016	-1.135	0.258	-0.051	0.014
Physical_health	0.042	0.040	1.055	0.293	-0.036	0.121
Mental_health	0.081	0.044	1.828	0.070	-0.006	0.167
Self_nature	0.020	0.028	0.710	0.479	-0.035	0.075
CyclingFreq	-0.051	0.028	-1.831	0.069	-0.105	0.004

SVS_change: change in pre-post mean scores of the Subjective Vitality Scale (Bostic et al., 2000; Ryan & Frederick, 1997)

	Estimate	Std. Error	t value	Pr(>|t|)	2.5 %	97.5 %
(Intercept)	0.306	0.602	0.508	0.612	-0.874	1.486
Environment_IDLow greenery	0.285	0.207	1.374	0.172	-0.122	0.692
Environment_IDHigh greenery	0.229	0.202	1.132	0.260	-0.167	0.625
Environment_IDWinter	0.116	0.204	0.571	0.569	-0.283	0.515
as.factor(Gender_Male)	-0.016	0.143	-0.114	0.910	-0.296	0.264
as.factor(Age_25_44)	-0.076	0.190	-0.399	0.690	-0.449	0.297
as.factor(Age_45_65)	0.096	0.256	0.374	0.709	-0.406	0.598
Stress_day	0.051	0.038	1.328	0.186	-0.024	0.126
Physical_health	-0.165	0.094	-1.758	0.081	-0.348	0.019
Mental_health	-0.133	0.103	-1.292	0.199	-0.335	0.069
Self_nature	0.103	0.066	1.573	0.118	-0.025	0.232
CyclingFreq	0.058	0.065	0.890	0.375	-0.070	0.185

SCL: Adjusted to baseline Skin Conductance Level (recovery period)

	Estimate	Std. Error	t value	Pr(>|t|)	2.5 %	97.5 %
(Intercept)	0.233	0.965	0.242	0.810	-1.659	2.125
as.factor(Environment_ID)2	0.484	0.335	1.445	0.151	-0.172	1.141
as.factor(Environment_ID)3	0.604	0.317	1.904	0.059	-0.018	1.225
as.factor(Environment_ID)4	0.057	0.321	0.177	0.859	-0.572	0.686
as.factor(Gender_Male)	-0.007	0.227	-0.030	0.976	-0.451	0.437
as.factor(Age_25_44)	-0.621	0.308	-2.014	0.046	-1.226	-0.017
as.factor(Age_45_65)	-1.147	0.405	-2.831	0.005	-1.940	-0.353
Stress_day	0.089	0.061	1.468	0.145	-0.030	0.209
Physical_health	-0.008	0.152	-0.053	0.958	-0.305	0.289
Mental_health	-0.192	0.168	-1.140	0.256	-0.521	0.138
Self_nature	0.226	0.103	2.191	0.030	0.024	0.428
CyclingFreq	0.073	0.106	0.690	0.491	-0.134	0.280

Blink rate (recovery period)

	Estimate	Std. Error	t value	Pr(>|t|)	2.5 %	97.5 %
(Intercept)	4.297	1.700	2.527	0.013	0.964	7.629
as.factor(Environment_ID)2	1.578	0.578	2.727	0.007	0.444	2.711
as.factor(Environment_ID)3	-0.018	0.559	-0.032	0.974	-1.113	1.077
as.factor(Environment_ID)4	-0.265	0.565	-0.469	0.640	-1.371	0.842
as.factor(Gender_Male)	-0.961	0.385	-2.493	0.014	-1.716	-0.205
as.factor(Age_25_44)	0.919	0.527	1.743	0.084	-0.114	1.952
as.factor(Age_45_65)	2.087	0.681	3.065	0.003	0.753	3.421
Stress_day	-0.087	0.104	-0.844	0.400	-0.291	0.116
Physical_health	0.367	0.266	1.380	0.170	-0.154	0.889
Mental_health	-0.378	0.289	-1.311	0.193	-0.944	0.187
Self_nature	-0.214	0.174	-1.230	0.221	-0.554	0.127
CyclingFreq	-0.049	0.180	-0.273	0.786	-0.401	0.303

Mean pupil diameter (recovery period)

	Estimate	Std. Error	t value	Pr(>|t|)	2.5 %	97.5 %
(Intercept)	4.958	0.665	7.457	0.000	3.655	6.261
as.factor(Environment_ID)2	0.400	0.226	1.771	0.079	-0.043	0.844
as.factor(Environment_ID)3	0.436	0.219	1.997	0.048	0.008	0.865
as.factor(Environment_ID)4	0.669	0.221	3.029	0.003	0.236	1.102
as.factor(Gender_Male)	-0.125	0.151	-0.831	0.408	-0.421	0.170
as.factor(Age_25_44)	-0.469	0.206	-2.275	0.025	-0.873	-0.065
as.factor(Age_45_65)	-0.766	0.266	-2.879	0.005	-1.288	-0.245
Stress_day	-0.027	0.041	-0.667	0.506	-0.106	0.052
Physical_health	-0.010	0.104	-0.100	0.921	-0.214	0.194
Mental_health	0.000	0.113	-0.001	1.000	-0.221	0.221
Self_nature	-0.041	0.068	-0.602	0.548	-0.174	0.092
CyclingFreq	0.059	0.070	0.846	0.399	-0.078	0.197

3. Variance partition plots for the regression models

We conducted variance partition analysis for each of the regression models presented above. We calculated the total R^2 for each model (quantifying the amount of observed variance in the response variable explained by the model) and the indidivual contributions of each covariate towards that. We used R-function glmm.hp for this. Below are the variance partition plots for each regression model, where the title indicates the response variable and the total R^2 captured.

Reference: glmm.hp: an R package for computing individual effect of predictors in generalized linear mixed models. Journal of Plant Ecology,15(6):1302-1307

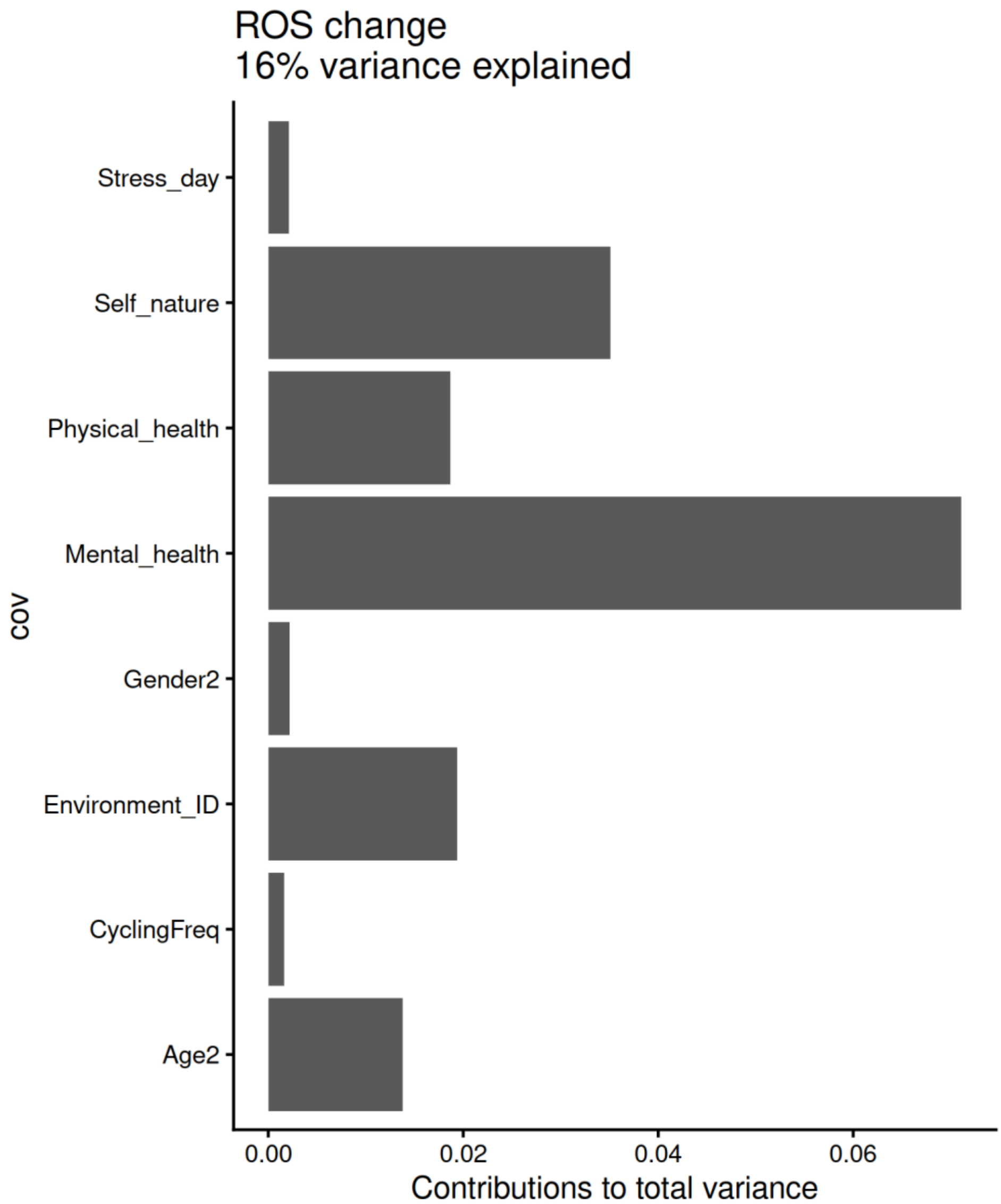

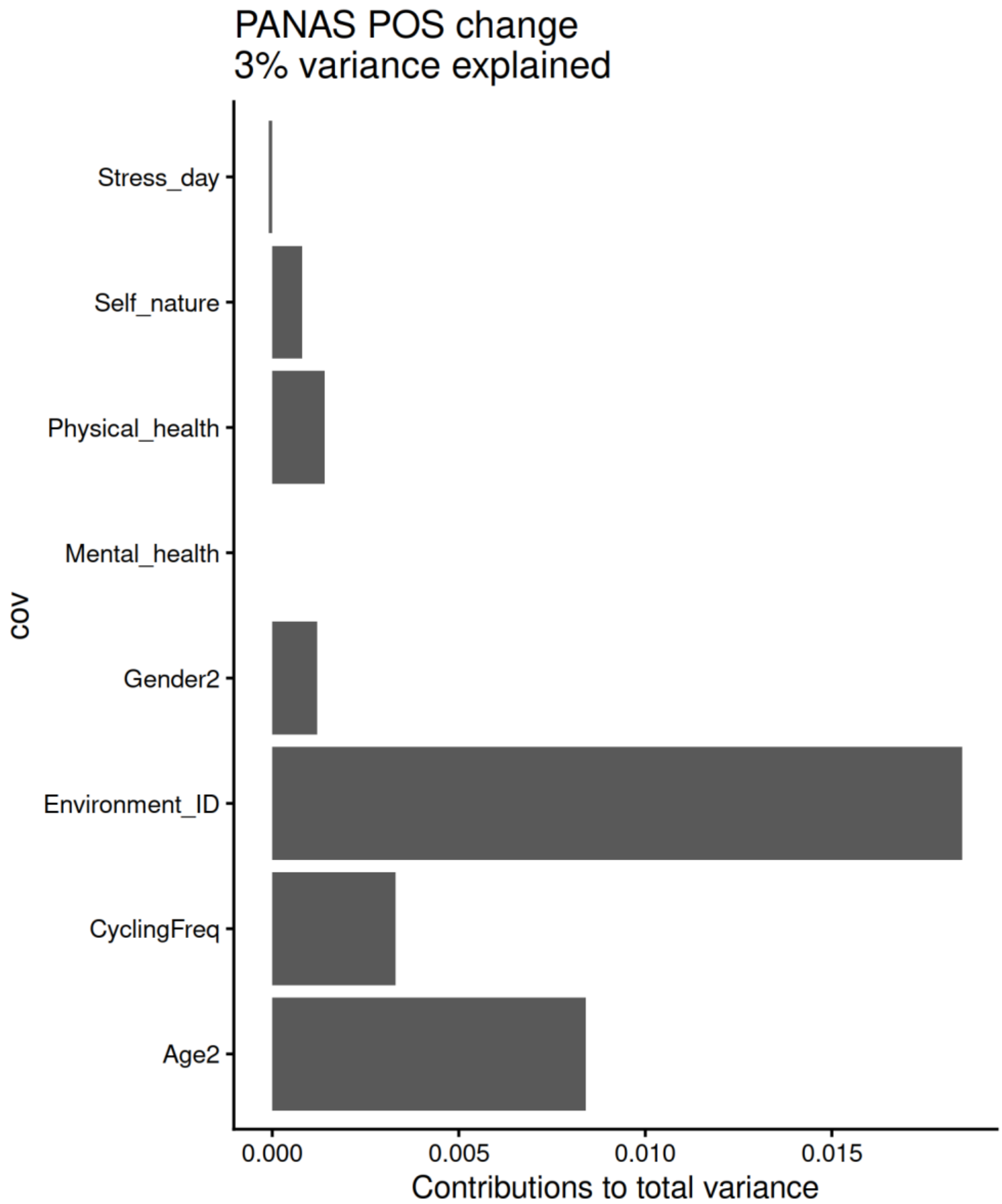

PANAS POS change
3% variance explained
Stress_day
Self_nature
Physical_health
Mental_health
Gender2
Environment_ID
CyclingFreq
Age2
cov
0.000
0.005
0.010
0.015
Contributions to total variance

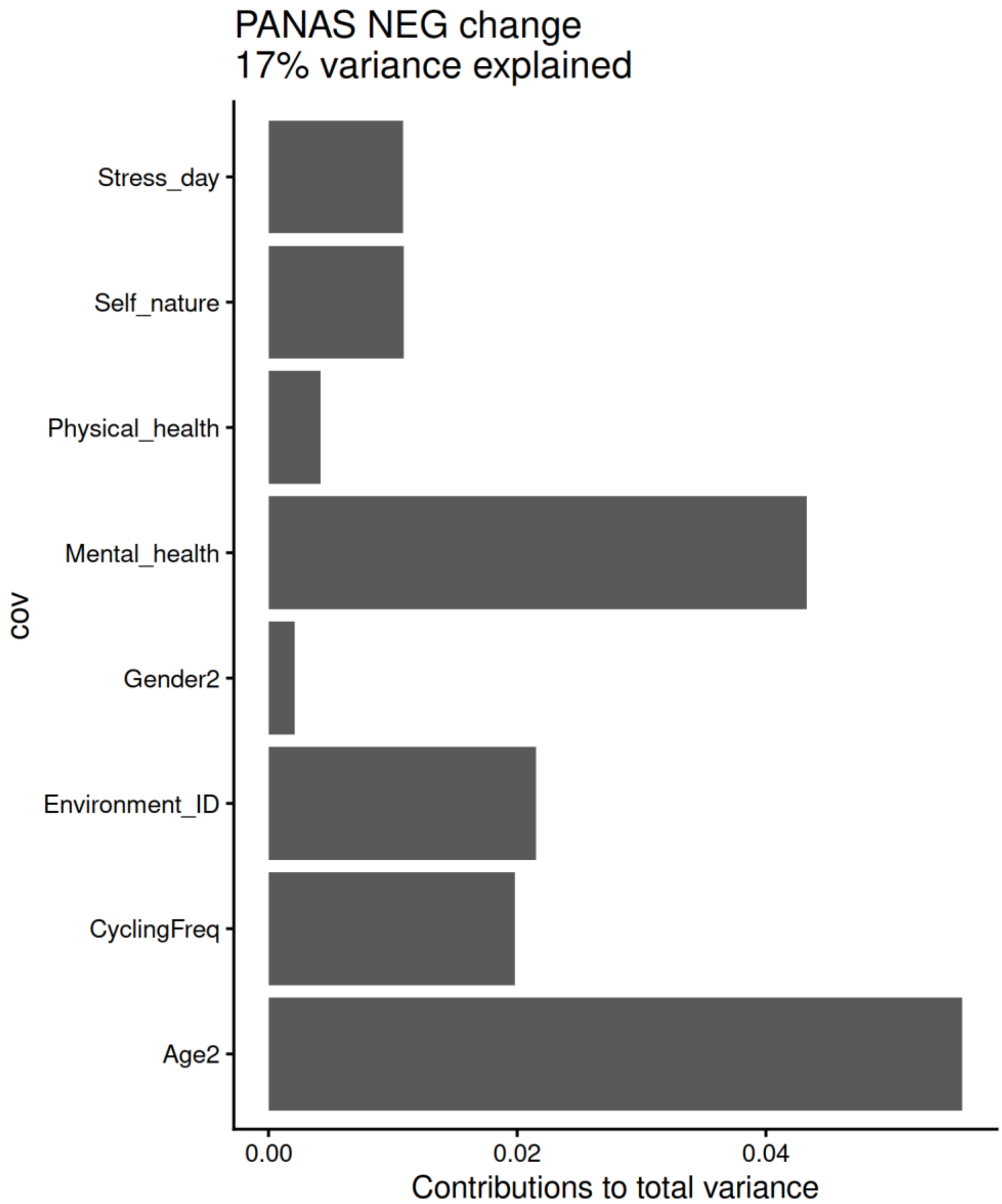
PANAS NEG change
17% variance explained
Stress_day
Self_nature
Physical_health
Mental_health
Gender2
Environment_ID
CyclingFreq
Age2
cov
0.00
0.02
0.04
Contributions to total variance

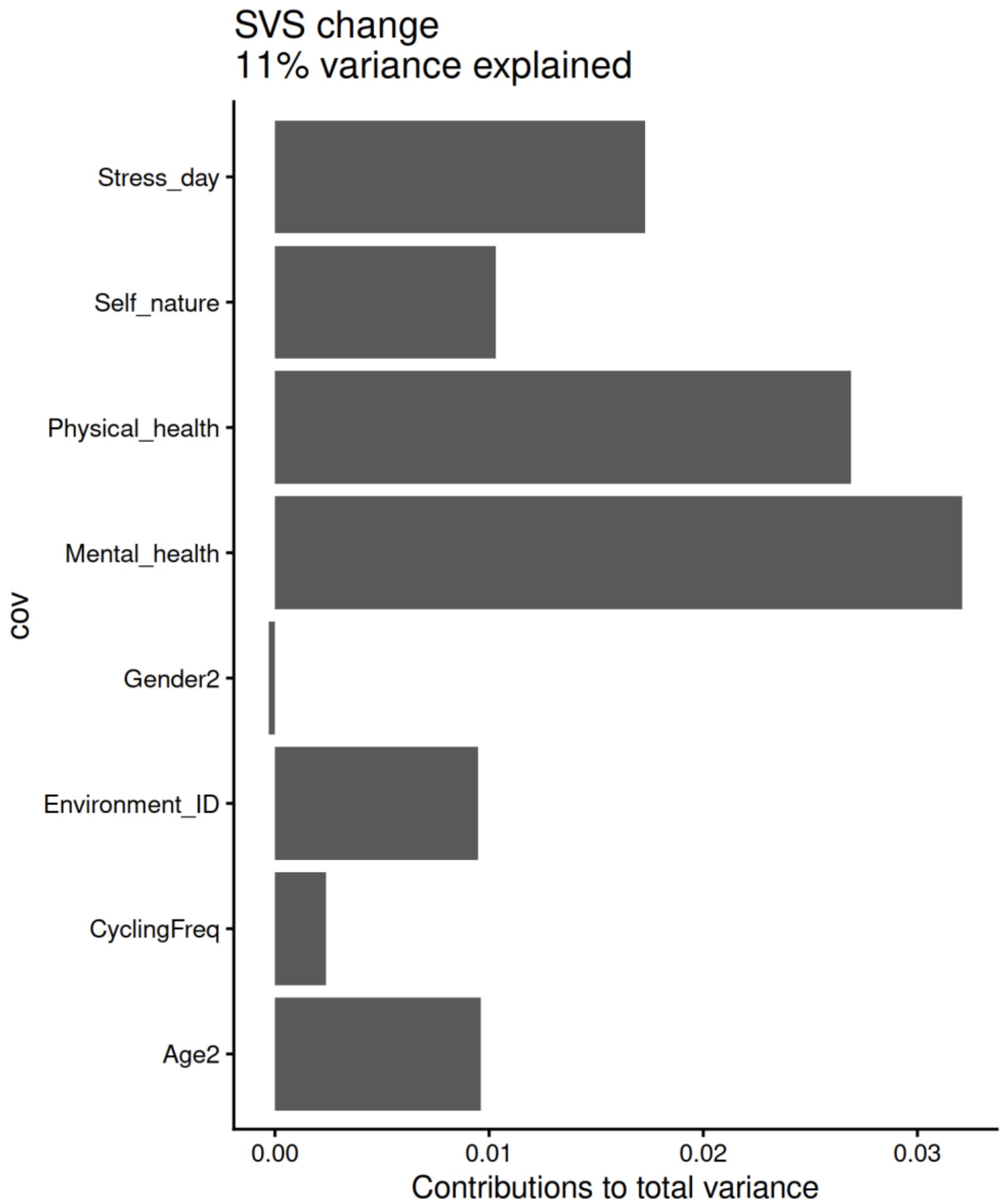

SVS change
11% variance explained
Stress_day
Self_nature
Physical_health
Mental_health
Gender2
Environment_ID
CyclingFreq
Age2
cov
0.00
0.01
0.02
0.03
Contributions to total variance

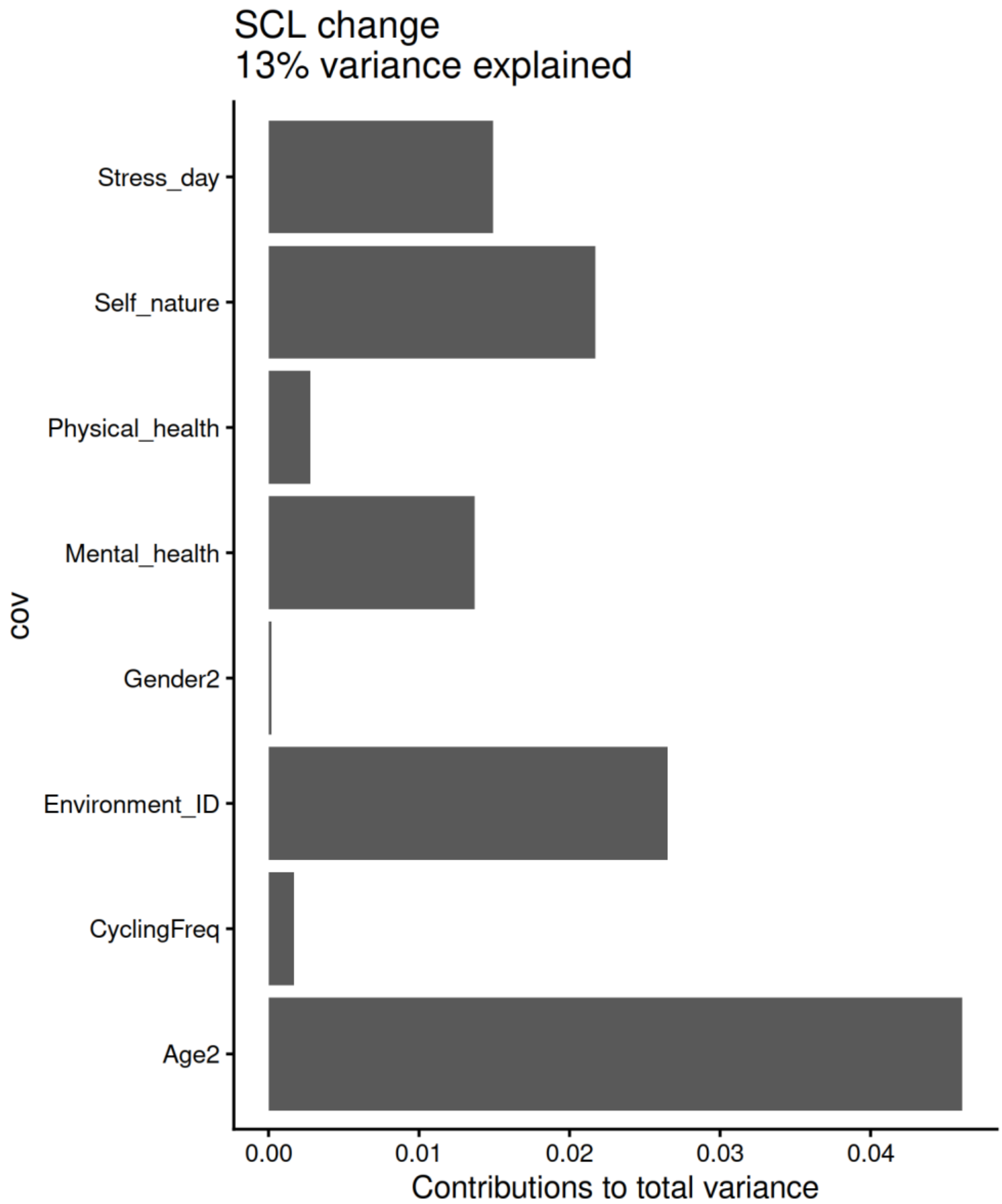
SCL change
13% variance explained
Stress_day
Self_nature
Physical_health
Mental_health
Gender2
Environment_ID
CyclingFreq
Age2
cov
0.00
0.01
0.02
0.03
0.04
Contributions to total variance

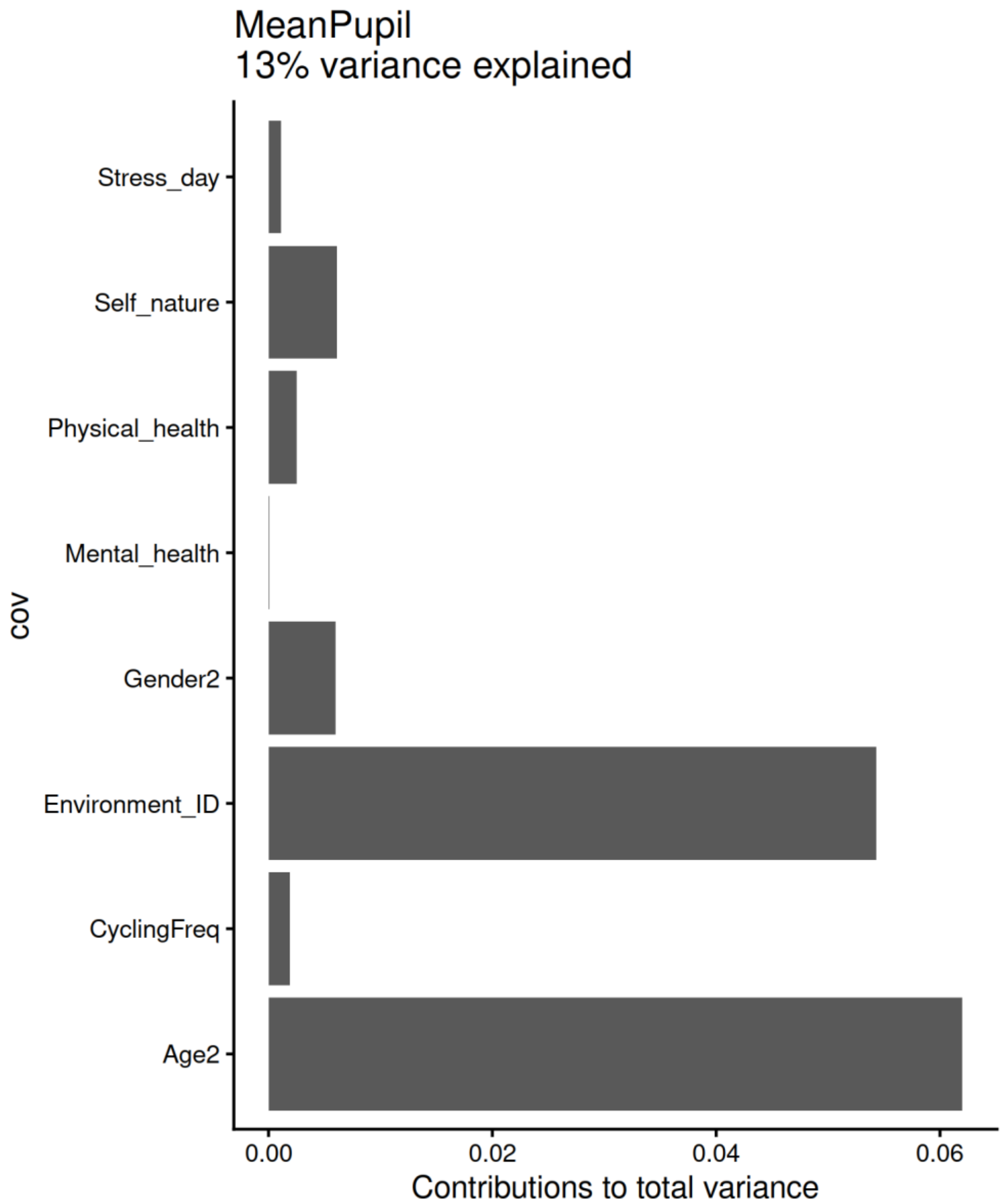
MeanPupil
13% variance explained
Stress_day
Self_nature
Physical_health
Mental_health
Gender2
Environment_ID
CyclingFreq
Age2
cov
0.00
0.02
0.04
0.06
Contributions to total variance

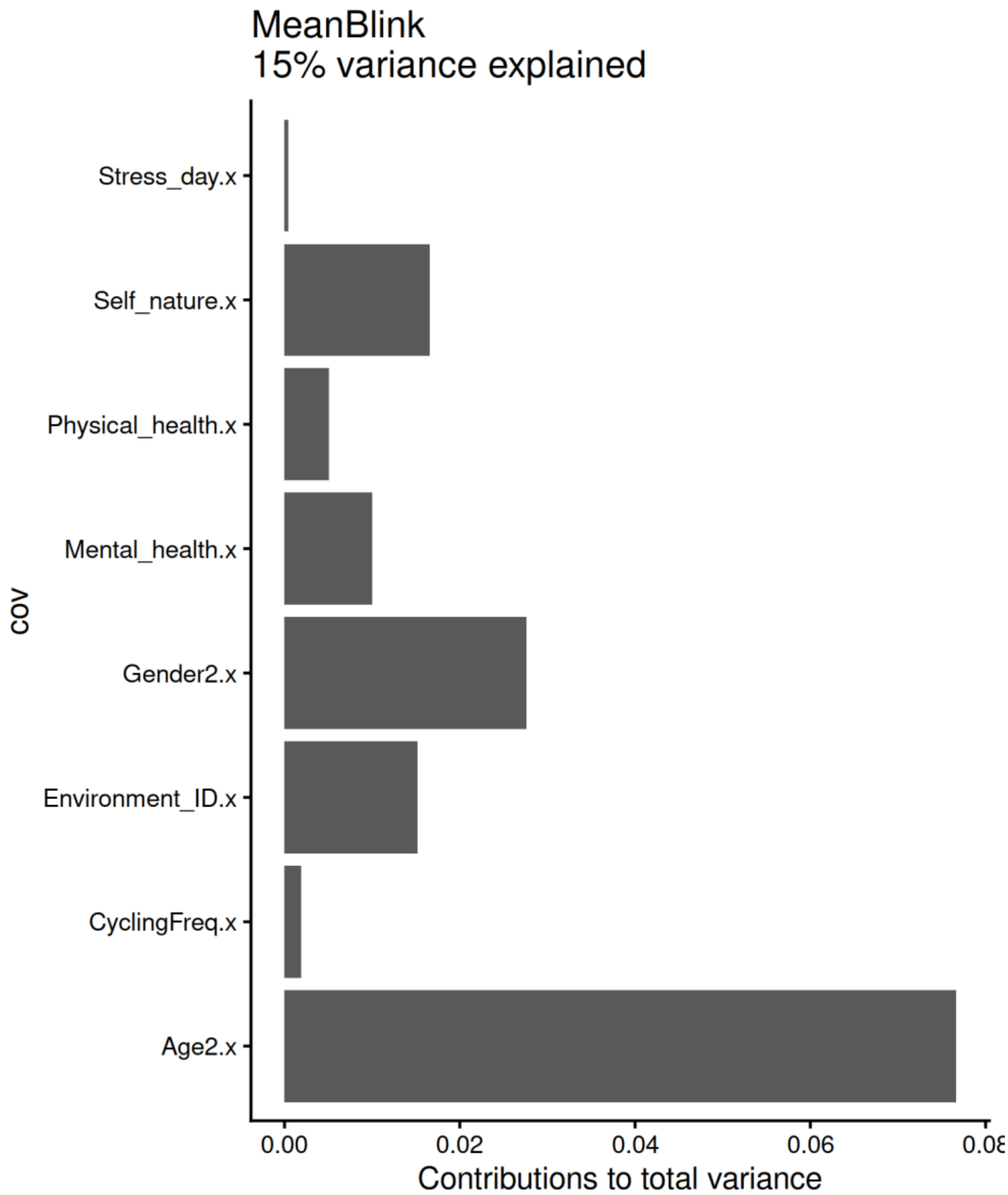

MeanBlink
15% variance explained
Stress_day.x
Self_nature.x
Physical_health.x
Mental_health.x
Gender2.x
Environment_ID.x
CyclingFreq.x
Age2.x
cov
0.00
0.02
0.04
0.06
0.08
Contributions to total variance
